# Road Traffic Injuries among Patients Visiting the Emergency Department in a Tertiary Care Centre: A Descriptive Cross-sectional Study

**DOI:** 10.31729/jnma.7895

**Published:** 2022-11-30

**Authors:** Alok Atreya, Arjun Kc, Samata Nepal, Ritesh George Menezes, Anuska Khadka, Palak Shah, Rakshya Arun Kandel

**Affiliations:** 1 Department of Forensic Medicine, Lumbini Medical College and Teaching Hospital, Tansen, Palpa, Nepal; 2 Department of Orthopedics and Trauma, Lumbini Medical College and Teaching Hospital, Tansen, Palpa, Nepal; 3 Department of Community Medicine, Lumbini Medical College and Teaching Hospital, Tansen, Palpa, Nepal; 4 Forensic Medicine Division, Department of Pathology, College of Medicine, Imam Abdulrahman Bin Faisal University, Dammam, Eastern Province, Saudi Arabia; 5 Lumbini Medical College and Teaching Hospital, Tansen, Palpa, Nepal; 6 Department of Internal Medicine, Sumeru Hospital, Pulchowk, Lalitpur, Nepal

**Keywords:** *automobiles*, *demography*, *Nepal*, *soft tissue injuries*, *traffic accidents*

## Abstract

**Introduction:**

Road traffic injuries are preventable yet one of the most neglected public health issues. Road traffic injuries not only impact the health of the victim but also cause financial burden to the entire family. This study aimed to find out the prevalence of road traffic injuries in patients visiting the Emergency Department in a tertiary care centre.

**Methods:**

A descriptive study was conducted among patients visiting the Emergency Department in a tertiary care centre from 1 January 2021 to 30 June 2021 after receiving ethical approval from the Institutional Review Committee (Reference number: IRC-LMC 07-J/020). Demographic information of the patients, accident profile and type of intervention at the hospital, and outcome were studied. Point estimate and 95% Confidence Interval were calculated.

**Results:**

Among 8,765 patients visiting the emergency department, road traffic injuries were seen in 112 (1.28%) (1.04-1.52, 95% Confidence Interval).

**Conclusions:**

The prevalence of road traffic injuries was found to be similar to other studies conducted in similar settings.

## INTRODUCTION

Road traffic injuries (RTIs) are one of the leading causes of Emergency Department (ED) admissions in Nepal.^1,2^ Road injuries ranked first among adolescents aged 10-24 years and also in the 25-49 years age group between 1990 and 2019 as per the global burden of disease and injury study in 204 countries.^3^ There is an increasing trend in the incidence of traffic injuries in Nepal in the past decade (2009-10 to 2019-20) with at least 15,554 cases reported in 2019 alone.^4^

In 2017, Nepal witnessed a total of 3,394 deaths related to transportation injuries out of 16,831 deaths related to injuries.^5^ The most commonly affected population in road-traffic accidents are the youth which will not only hamper the quality of life but also the economic growth of the country.^6^ Strategic action plans and awareness among the youths would see a decline in the number of traffic-related accidents.

This study aimed to find out the prevalence of road-traffic injuries in patients visiting the ED in a tertiary care centre.

## METHODS

A descriptive cross-sectional study was conducted on the cases of road traffic injuries visiting the ED of Lumbini Medical College (LMC) and Teaching Hospital, Palpa, Nepal. The study was conducted from 1 January 2021 to 30 June 2021 after obtaining ethical approval from the Institutional Review Committee (Reference number: IRC-LMC 07-J/020) of LMC. The study included all the cases of road traffic injuries reported and admitted during the study period and excluded patients who were dead when they presented to the ED even though the history suggested road-traffic accidents. The sample size was calculated using the formula:


$$\begin{array}{ll} \text{n} & ={\text{Z}}^{2}\times \frac{\text{p}\times \text{q}}{{\text{e}}^{\text{2}}}\\ & =1.96^{2}\times \frac{0.056\times 0.944}{0.01^{2}}\\ & =2031 \end{array}$$

Where,

n= minimum required sample size
Z= 1.96 at 95% Confidence Interval (CI)
p= prevalence of RTI, 5.6%^2^
q= 1-p
e= margin of error, 1%

On quadrupling the sample size, it becomes 8124. However, the final sample size taken was 8765.

The information about the patients was obtained daily from the ED as all the cases of RTIs present directly to the ED. Informed consent was obtained from the patients. In cases where the patients could not provide consent because of their underage, psychiatric illness or impaired consciousness, the consent was obtained from their proxy. The information obtained was the type of vehicles involved in the crash, the experience of the driver, time of the day of the incident, type of impact, whether first aid was received or not, how the patient was brought to the hospital, the time of arrival to the hospital and the Glasgow coma scale (GCS) on arrival. The in-patients were then followed up once daily for the investigations, the diagnosis, and treatment/intervention until the outcome was reached. All the collected information was noted in a standard proforma.

Data thus collected were entered and analysed using IBM SPSS Statistics version 25. Point estimate and 95% CI were calculated.

## RESULTS

Among 8,765 patients visiting the emergency department, road traffic injuries were seen in 112 (1.28%) (1.04-1.52, 95% CI). There were 74 (66.07%) males and 38 (33.93%) females with a male to female ratio of 1.95:1. The victims involved in the accident were aged from 3 years to 76 years with a mean age of 29.70±16.50 years. The place of accident was Palpa district in the majority of the cases 75 (66.96%) followed by Gulmi 25 (22.32%) with a few cases from Arghakhanchi, Parbat, Baglung, Syangja, Pyuthan, and Rupandehi. Two-wheelers were the most commonly involved vehicles in the RTIs in the present study in 52 (46.43%) followed by 43 (38.39%) 4-wheelers. When the victim profile was evaluated it was observed that 43 (38.39%) were passengers and 27 (24.11%) pedestrians (Table 1).

**Table 1:** Demographic details of the patients (n= 112).

Variables		n (%)
**Gender**	Male	74 (66.07)
	Female	38 (33.93)
**Type of vehicle involved**	2-wheeler	52 (46.43)
	3-wheeler	7 (6.25)
	4-wheeler (car, van, jeep)	43 (38.39)
	Passenger bus	2 (1.79)
	Truck/tipper	1 (0.89)
	Tractor	2 (1.79)
	Other	5 (4.46)
**Victim profile**	Pedestrian	27 (24.11)
	2-wheeler rider	25 (22.32)
	2-wheeler pillion rider	13 (11.61)
	Driver	4 (3.57)
	Passenger	43 (38.39)
**Driving/riding experience**	Less than 5 years	9 (8.04)
	5-10 years	8 (7.14)
	11-15 years	9 (8.04)
	16-20 years	3 (2.68)
	Not applicable	83 (74.11)

**Figure 1:**
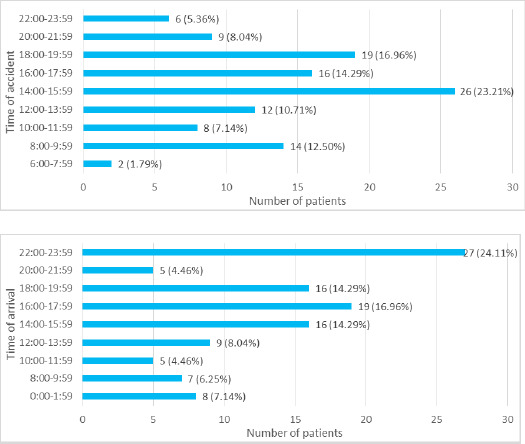
Time of accident and time of arrival of the patients to the hospital (n= 112).

Time of arrival to the hospital after the accident ranged from 00:30 hour to 20:18 hours with a mean duration of 3:44±3:25 hours (Figure 1).

The head-on collision was the most common type of vehicular impact. A total of 55 (49.11%) patients used an ambulance to reach the ED while 57 (50.89%) did not wait for the ambulance and rushed to the hospital in the available vehicle (Table 2).

**Table 2:** Characteristics of road traffic accidents (n= 112).

Variable		n (%)
**Type of vehicular impact**	Head-on collision	71 (63.39)
	Side impact	30 (26.79)
	Rear end collision	11 (9.82)
**Transportation used to reach the ED**	Ambulance	55 (49.11)
	Other than ambulance	57 (50.89)
**Primary care before arrival**	Received	26 (23.21)
	Not received	86 (76.79)

When the site of injury was evaluated it was seen that 22 (19.64%) had more than one region of the body injured. The X-ray was done in 68 (60.71%). There were no cases where the GCS score was below 9 (Table 3).

**Table 3:** Injury profile (n= 112).

Variables		n (%)
**Site of injury**	Head, neck and face	16 (14.29)
	Spine	3 (2.68)
	Upper extremities	30 (26.79)
	Lower extremities	31 (27.68)
	Thorax	7 (6.25)
	Abdomen	2 (1.79)
	Pelvic region	1 (0.89)
	More than one region involved	22 (19.64)
**Investigations done**	X-ray	68 (60.71)
	CT	11 (9.82)
	MRI	1 (0.89)
	USG	1 (0.89)
	X-ray and CT	12 (10.71)
	Multiple	18 (16.07)
	None	1 (0.89)
**Type of injury**	Head injury	2 (1.79)
	Injury to spine	3 (2.68)
	Fracture of bones	13 (11.61)
	Dislocation	2 (1.79)
	Soft tissue injury	52 (46.43)
	Soft tissue injury and fracture of bones	22 (19.64)
	Head injury, soft tissue injury and fracture of bones	9 (8.04)
	None	9 (8.04)
**GCS on arrival**	Mild (13-15/15)	111 (99.11)
	Moderate (9-12/15)	1 (0.89)

The Department of Orthopaedics and Trauma was consulted for all the cases. Orthopaedics and Neurosurgery consultation was done in 14 (12.50%) cases and multidisciplinary consultation was done in 15 (13.39%) cases. There were 4 (3.57%) cases during the study period that were referred to other hospitals. The present study reported 5 (4.46%) cases who were discharged upon request and 2 (1.79%) cases who discontinued treatment and left against medical advice (Table 4).

**Table 4:** Treatment profile (n= 112).

Variables		n (%)
**Consulting departments**	Orthopedics only	82 (73.21)
	Orthopedics and Neurosurgery	14(12.50)
	Orthopedics and General Surgery	1 (0.89)
	Multidisciplinary	15(13.39)
**Intervention by orthopaedics**	No intervention required	5 (4.46)
	Local wound care	54 (48.21)
	Closed reduction and cast application	24 (21.43)
	Open reduction	7 (6.25)
	Admission and observation	11 (9.82)
	Admission and multiple interventions	11 (9.82)
**Treatment outcome**	Discharged with advice	101 (90.18)
	Discharged on request	5 (4.46)
	Referred to other hospitals	4 (3.57)
	Left against medical advice	2(1.79)

## DISCUSSION

We found that more males were involved in traffic accidents than females and the time of day was afternoon hours when many of these accidents happened. The majority of the victims were middle-aged with a mean age of 29.70±16.50 years. The mean time of arrival to the hospital after the accident was 3:44±3:25 hours. Soft tissue injuries were more common injuries followed by fractures of bones. The majority of the patients were discharged after treatment. We also found that two-wheelers were the most common type of vehicle involved in the accidents which is a similar finding when compared to other studies.^2,7-11^ In 2018, RTI was the leading cause of death among people aged 5-29 years globally, who were either pedestrians or two-wheeler riders from low-income countries.^12^ Various studies have also reported that male youngsters are more involved in RTIs when compared to females.^8-11,13^

The number of patients who arrived at the ED in an ambulance was almost equal to the number of patients who reached the ED in other vehicles. However, the number of patients receiving first aid before reaching the tertiary care hospital was fairly less. It may be due to the lesser number of primary health care centres (PHCC) in rural areas. The other reasons might be due to a lack of medical doctors in these PHCCs and also a lack of basic screening equipment like X-ray machines.^14^

When the location of the injuries was evaluated it was observed that the majority of the injuries were on the extremities. There were very few cases of thoracoabdominal injuries and head injuries. Most of the injuries were in the form of soft tissue injuries in the present study which is similar to other studies from Nepal.^2,13^ Except for a single case, all cases in the present study had either normal or mildly impaired GCS scores. However, when autopsy-based studies with fatal outcomes are reviewed, head trauma and thoracoabdominal injuries are more commonly reported.^14^

There is a compulsory law in Nepal that a rider should wear a helmet with straps on it. But there is no mandate on the type of helmet that should be worn. With the availability of different types of designer helmets, half helmets and full helmets (with visor and neck protection) the riders use them for compulsion and fashion rather than protection. There is a law for two-wheeler pillion riders too to wear a helmet, however, pillion riders are seldom punished for not wearing one.^15^ Due to regular checking by the traffic police, the riders in the urban areas follow the compulsory helmet use. However, rural location riders ply the road without a helmet.^15^ There were cases in the present study where riders sustained injuries on the face due to broken pieces of visor while wearing a half helmet. Similarly, one patient in the present study who sustained facial injuries was referred to a higher centre for facial reconstruction who had not worn a helmet during the accident.

Road traffic accidents and subsequent injuries not only add pain to the victim but also add a financial burden to the family.^16^ There are laws mandated by the government for road traffic safety e.g. compulsory helmet use for motorbike riders, compulsory seat belt use, no tolerance in drinking and driving, etc., but due to lack of awareness, these are not strictly followed. A study conducted in lower-middle-income countries reported limited and relatively weak evidence regarding cost-effective interventions to prevent RTIs.^17^ The rules imposed by the government are taken as compulsion and burden by the drivers/riders rather than a safety measure.

The major limitation of the present study is that it did not estimate the economic cost due to RTIs. Furthermore, the education level and socioeconomic status of the patient were also not considered. Education plays a vital role in the reduction of traffic-related accidents. It is recommended that road safety and traffic rules be incorporated into the school curriculum to educate young children. The government also should make a policy of 'sound vehicle on the road' and also instigate regular vehicle examination and driving license.

## CONCLUSIONS

The prevalence of road traffic injuries among patients visiting the Emergency Department was found to be similar to other studies conducted in similar settings. Most of the factors responsible for traffic accidents and their consequences are preventable. The youth should be made aware that the traffic rules should be followed for their own safety rather than compulsion. Incorporating traffic rules and safety in the school curriculum will help minimize traffic accidents in the long run in Nepal.
